# The role of philosophy and ethics at the edges of medicine

**DOI:** 10.1186/s13010-021-00114-w

**Published:** 2021-11-06

**Authors:** Bjørn Hofmann

**Affiliations:** 1 Department of Health Sciences, The Norwegian University Science and Technology, Gjøvik, PO Box 1, N-2802 Gjøvik, Norway; 2 Centre for Medical Ethics at the University of Oslo, Oslo, Norway

**Keywords:** Edge, Metaphor, Role, Ethics, Philosophy, Challenges, Technology

## Abstract

**Background:**

The edge metaphor is ubiquitous in describing the present situation in the world, and nowhere is this as clearly visible as in medicine. “The edge of medicine” has become the title of books, scholarly articles, media headlines, and lecture series and seems to be imbued with hype, hope, and aversion. In order better to understand what is at stake at “the edge of medicine” this article addresses three questions: What does “the edge of medicine” mean in contemporary debates on modern medicine? What are the challenges “on the edge of medicine” (in these various meanings of “on the edge”)? How can philosophy and ethics contribute with addressing these challenges?

**Methods:**

Literature searches in PubMed and Google Scholar are used to identify uses of the phrase “the edge of medicine” while conventional content analysis is used to analyze meanings of and challenges with “the edge of medicine.” These results are then investigated with respect to how philosophy and ethics can address the identified challenges.

**Results:**

The literature reveals that “the edge of medicine” has many meanings, such as: Border; Margin (of life); Frontier; Forefront; Fringes; Plunge (abyss); Brink (verge); Conflict; and Balancing. In general, the various meanings address four basic challenges: setting limits, keeping control, make meaning, and handling conflicts or aporias. The analysis of each of the meanings of “the edge of medicine” identifies a wide range of important and urgent tasks for the humanities in general, and for philosophy and ethics in particular: 1) clarifying concepts; 2) clarifying assumptions and premises of arguments, methods, advice, and decisions; 3) elaborate new concepts and new theories; 4) conceptualize and handle uncertainty, moral regret, and residue; 5) reveal “the emperor’s new clothes;” 6) identify trends and reflect on their implications; 7) demarcation; and 8) reflecting on goodness in medicine.

**Conclusion:**

The phrase “the edge of medicine” expresses a wide range of challenges for modern health care. Together with other disciplines philosophy and ethics can and should make crucial contributions at “the edge of medicine,” which is where the future of human beings and societies is created and formed.

## Background

In their book “On the edge” from 2000 Hutton, Giddens, and Meyers claim that the significant changes of new technologies and globalization has put the world on the edge [1]. Twenty years later, this conception of a world on the edge is strongly enhanced. Nowhere is this more vigorously experienced than in medicine, where gene editing, gene drives, Big Data, artificial intelligence, organoids, and chimeras provide unprecedented possibilities but also unforeseeable implications. Moreover, medicine, in the wake of the magic-bullet metaphor, is rapidly expanding its subject matter and the need for medical services is skyrocketing.

This very development raises questions about the essence and goal of medicine: What should medicine do? Whom should it serve? These questions and the topic at “the edge of medicine” has been on the agenda of books [2, 3], scholarly articles [4–6], media coverage [7],^1^ lecture series (Ernest Beutler Lecture on the Edge of Medicine),^2^ and conferences (ESPMH in Oslo 2019). Terms such as on or at “the edge of medicine” and “medicine on the edge of life” are frequently used in the literature [8]. Hence, the conception of being at the edge appears to be pervasive and raises the question of what it means to be at “the edge of medicine” and what is the role of the humanities at this edge.

As the task of investigating all humanities at “the edge of medicine” in general is too comprehensive, this study will be limited to the role of philosophy and ethics. As the phrasing “the edge of medicine” seems to have many meanings, I will start with investigating different meanings of the claim that medicine is on the edge. Then I will analyze what challenges are related to these meanings. Lastly I will investigate the role of philosophy in general, and ethics in particular in addressing these challenges.

## Methods

The aim of this study is to investigate the challenges with being on “the edge of medicine” and how philosophy and ethics can contribute to address these challenges. Accordingly, this article will address three questions:

1. What does “the edge of medicine” mean in contemporary debates on modern medicine?
2. What are the challenges at “the edge of medicine” (in these various meanings of at “the edge”)?
3. How can philosophy and ethics contribute with addressing these challenges?

Literature searches in PubMed and Google Scholar were used to identify uses of the phrasing “the edge of medicine.” Supplementary snowball searches were performed, but were stopped when the searches did not add new meanings to the phrase or affiliated challenges. The references were investigated with conventional content analysis [9] for a) meanings of “the edge of medicine” and b) affiliated challenges to medicine. These results were then investigated with respect to how philosophy and ethics could address the identified challenges.

## Results

### What does “the edge of medicine” mean, what are the challenges, and how can philosophy and ethics contribute?

The literature reveals that there are many meanings of the phrase “the edge of medicine.” These meanings can be subsumed under the headings: border, margin, frontier, fringe, brink, conflict, plunge, and as “balancing on the edge.” In the following I will present some of these meanings and give examples, starting with “the edge of medicine” as a border.

#### Edge as border

One of the frequently referred meanings of “the edge of medicine” seems to be “the border of medicine” in terms of what belongs to or counts as medicine (is inside) and not (i.e., what is outside). This understanding can be identified in the many vivid debates on what counts as a disease. In the recent revisions of the Diagnostic and Statistical Manual of mental disorders (DSM) and the International Classification of Diseases (ICD) there have been fierce debates on whether specific conditions, such as Aspergers syndrome [10, 11] and gender incongruence [12–14] should count as diseases and be diagnoses. Correspondingly, there are extensive debates on the disease status of obesity [15–27] and ageing [28–33]. The key issue is the inside-outside question.

Similar cases can be identified on the border between aesthetics and ethics, e.g., in cosmetic surgery [34]. Here the key question is whether cosmetic surgery (beyond regeneration) is *belonging to the tasks* of medicine or not [35]. Male circumcision is a related example where the question of whether this should be provided and covered by the health services in countries with universal coverage in health services is heavily debated. The role and responsibilities of patients and proxies in the case of home treatment of patients is yet another example. Relatives of patients with Amyotrophic lateral sclerosis (ALS) may be heavily involved in their treatment and care. However, are there limits to their efforts and responsibility? These and similar questions ask for the borders of medicine. What is outside and what is inside?

Another case of epistemic interest is the ability to expand the conception of disease beyond what is verifiable in terms of human suffering and disability [36–38]. One example of this is overdiagnosis, which is defined as diagnosing conditions that would never have bothered the person if they were not detected [39]. For a wide range of screening programs we are able to find risk factors, predictors, or precursors of diseases. However, we do not know whether what we correctly find (a true positive test result) will ever develop into symptoms or disease [40]. Nonetheless, in order to avoid the situations where they do, we treat them all. Thereby we come to label and treat a great number of people as diseased appropriately. In our eager of doing something good we may end up doing too much, and in sum, doing more harm than good [39, 41].

Another hot and “edgy” topic is social freezing of gametes (eggs) without medical indication. Should this be a task of medicine and health care or not? [42] Where are the borders of health care?

The key issue for the interpretation of “the edge of medicine” as *border* is that of demarcation, and more specifically *the demarcation of the subject matter of medicine*. The main challenges are to differentiate between what is disease (illness or sickness) and not; what belongs to the goals and tasks of the health professional and the health care system and what is more appropriately handled by others; and when the health services do less good than harm.

How can philosophy and ethics contribute with respect to these challenges? One overarching and important, but difficult task, is defining the essence or goal of medicine. A clear conception of the core or goal of medicine could be very helpful for defining the borders of medicine. Attempts have been made [43–46] and been criticized [47, 48]. While no solution is yet obtained, the contributions are crucial.

Another important task of philosophy is to clarify basic concepts, such as disease, illness, sickness, diagnosis, aging, autonomy, coercion, etc., as a wide range of the border-making-challenges mentioned above result from the lack of conceptual clarity. One example of a fruitful contribution here worth mentioning is conceiving illness as uncanniness, e.g., as inspired by the study of Freud and Heideggers’ conception of “Unheimlichkeit.” [49].

Moreover, philosophy can contribute to the demarcation task by defining, analysing, identifying and targeting concepts such as diagnostic creep, overdiagnosis, overtreatment, and medicalization. One example is how important it is to analyse the context of diagnosis process and all sources of information when defining disease [50].

Additionally, philosophy and ethics can clarify the relationship between professionalism and ethics, between aesthetics and ethics, between medical and non-medical tasks. That is, to draw the line for the role of the medical profession.

What many of these borderline-drawing tasks boil down to is demarcating what is beneficial from what is harmful. Accordingly, what lies at the core of the task of demarcation is the issue of defining *medical goodness* [51] – an obvious, but difficult, task for philosophy in general and ethics in particular. This is clearly not the place to solve all these issues. The task of this article is more to provide an overview of the challenges at “the edge of medicine” and indicate where philosophy and ethics can make valuable and much needed contributions.

#### Edge as margin

Another and related use of “the edge of medicine” is edge as a margin, and in particular, as the tasks of medicine at the margins of life.

Decisions at the beginning and at the end of life are typical examples of such margins, and palliative care, withholding and withdrawing treatment, physician assisted suicide (PAS), and euthanasia can serve as examples of related issues. Questions of at what gestational age we should start to save foetuses or children at preterm births, are other challenging examples [52]. In research, when to allow research on and destruction of embryos has been has been a key question [53].

Basic philosophical questions are “when does life start?” What constitutes personhood? When does it end? What is, and what decides, moral status? Such questions call for metaphysical reflection but also for reflections on marginality and liminality [54] as well as vulnerability [55, 56].

The key issue in this conception of “the edge of medicine” is (ontological) demarcation and defining the tasks of medicine at the margins of life. Many of the basic questions are metaphysical and concern the content of key concepts, such as life, death, person, pain, pleasure, consciousness, moral status, vulnerability etc. Hence, while this has posed fundamental tasks for philosophers and ethicists for decades already, new options of extending life make them even more crucial.

#### Frontier

A prevalent understanding of “the edge of medicine” is as being at the frontier of medicine [2]. Advances in science and technology have significantly influenced medicine and provides tremendous and unprecedented opportunities. However, the potential also raises basic questions of how to develop, implement and apply these opportunities for the benefit of individuals, societies, and mankind.

BigData, AI, and Direct-to-Consumer (DtC) genetic testing are but some examples where the edge of medicine is used as a metaphor for frontier. Correspondingly, immune therapy and person-adapted treatments provide perplexing opportunities at the frontiers of treatment while gene editing and gene drives push the frontiers of (human) enhancement.

As a wide range of promising experimental treatments become available at the cutting “edge of medicine”, there are challenges with validation in ordinary robust studies (avoiding potential bias) [57]. This raises profound questions of reproducibility and verifiability, where philosophy can play a contributory role in clarifying conceptual, epistemic, and ethical issues.

For example, AI-based diagnosis and treatment decisions pose questions of validity and responsibility [58]. Medical enhancements raise issues of equity, justice, how to assess future benefits and harms, and how to set limits. They call for reflections on naturalness, on the therapy-enhancement-distinction, on the relationship between health and disease, and on the basic conception of goodness in medicine [59, 60].

Hence, the phenomena that are described as the frontiers of medicine raise a wide range of issues, such as altering the methods for knowledge production and validation, expanding the tasks and responsibilities of medicine, defining and demarcating human from non-human, natural from non-natural, therapy from enhancement, health from disease, medicine from pseudo-medicine etc. Additionally we face problems of cost containment, resource allocation, equity, and just distributions and access to care. In all these issues, philosophy and ethics can and are making important contributions and more are needed.

Most prominently, the “frontiers of medicine” challenge philosophy in the most profound way. While the core question for philosophy has been “what is a human being?” for thousands of years new technologies have reframed the question to “what does it mean to be a human being?” or “what should a human being be?” – not only as a social and cultural being, but as a biological organism and a biomolecular composition.

#### Fringe

Another important meaning of “the edge of medicine” is fringe. There are a wide range of situations in medicine and health care that are in what is often called “the grey zone,” i.e., where there are no clear conceptions of the situation or the rules and regulations that apply. In psychiatric the relationship between coercion and compulsion is but one example of such grey zones [61].

A wide range of alternative medicine is considered to be on the fringes of medicine. Historically alchemy has been considered to be mainstream medicine, now it is not [62]. The same goes for quackery and medical charlatanism [63]. Moreover, we face with the task of reversing the application of unuseful or futile treatments [64] and disinvest in low-value care [65].

The key tasks for philosophy and ethics to address at these fringes of medicine are: 1) avoid unnecessary conceptual vagueness (provide clear definitions of key concepts), 2) epistemic clarification (clarify the basis for warranted knowledge production), 3) ascertain moral relevance, and 4) provide demarcation of what belongs to medicine and what does not.

#### Plunge

Another crucial meaning of “the edge of medicine” is plunge or abyss. There are a wide range of situations in medicine and health care that cannot be resolved or that have no solutions. True and hard dilemmas prevail in health care, and there are ample situations in which doing the morally right thing is impossible [66, 67]. Correspondingly, moral residue is prevalent in clinical medicine as in health policy making [68] and mistakes are unavoidable [69] causing moral distress [70].

Accordingly, the understanding of “the edge of medicine” as a plunge is addressing challenges for professionals and is exemplified by moral distress, residue, remorse and regret, which is found among health professionals [71, 72], in education and training [73], as well as in management and amongst providers [74].

The task of philosophy of ethics in this understanding of “medicine on the edge” is among other things to define and support professionals in handling situations, such as culpability, shame, and blame; moral distress; residue, regret, and remorse; as well as tragic choices.

#### Brink

Related to the fringes of medicine, “the edge of medicine” is sometimes understood as being on the brink or verge of medicine. Modern western biologically oriented technologically advanced medicine has become a forceful paradigm of medicine in general. However, this paradigm does not fit for all settings and for all health care systems [75].

Moreover, in a globalised world we experience a wide range of global health emergencies. Ebola, avian flu, and SARS-COV-2 are but some examples where medicine is brought to its edge (both ethically and epistemically).

Correspondingly, the increase in migration and number of refugees and asylum seekers needing health services pose a wide range of ethical issues: access to care, DNA-testing, age determination in refugee children; domestic violence among asylum seekers.

The key issue in these quite diverse cases seems to be *relevance* and *jurisdiction*. Are our approaches in medicine relevant for other contexts, and how far does the jurisdiction of our health care system go?

What then are the contributions by philosophy and ethics in such cases? One important task is to reflect on cultural and social contingency and the universality (global applicability) of our approaches. Another difficult task is demarcation as well as reflecting on the goals of medicine. Emergency and disaster bioethics [76] are important contributions amongst others.

#### Conflict

Conflict is yet another important understanding of “the edge of medicine,” and can be envisioned as the edge between pressing (continental) plates. Conflicts can result from different perspectives and moral failure, and oftentimes result in moral dilemmas. They are characterized by moral disagreement, which can be studied in debates between so-called “bioconservatives” and “bioliberals” [77] where there seems to be little real communication or mediation.

The key challenge in this meaning of edge is disagreement and conflicting perspectives and the task of philosophy and ethics is to describe and clarify the conflicts and their premises, i.e., a kind of philosophical plate tectonics, but also to critique the arguments and perspectives, and of course, where possible to convey and negotiate. Another task is to analyse and classify the conflicts, e.g., in those that may be resolvable from those that are not [78].

#### Balancing on a blade

The last conception of “the edge of medicine” that will be discussed in this article is the notion of physical edge where you need balancing. Medicine in general and health professionals in particular, have to balance between a range of conflicting interests or between unacceptable situations or conditions.

Mammography (and other types of) screening can serve as an example, where balancing between polarized research findings [79] (as well as opposing strong interests and opinions) is a challenging task for health policy makers, health professionals, the general public, and women invited to screening to understand and make informed choices.

On a more global level, there are tremendous tasks with balancing optimism and pessimism; progress and regress; advancement and access in medicine and health care (as in food and education), and where medicine apparently is balancing on an edge. On the one hand we praise progress, reason, and enlightenment [80], but on the other hand we face huge challenges with making the results of this progress available to all people now and in the future.

The key issue in such cases is *judgement*, *balancing*, and *quality optimization*. Here philosophy and ethics can make major contributions in revealing, analysing, and balancing interests, perspectives and concerns [81].

### Some basic concerns behind «the edge of medicine»

Hence, “the edge of medicine” can mean many things, such as *border*, *margin*, *frontier*, *fringe*, *brink*, *conflict*, *plunge*, and as “*balancing on the edge*.” These may be grouped in four main categories according to the concern they express:

1. the borders of medical practice (border, margin, brink);
2. lack of control over areas or technologies (frontier);
3. lack of or altering meaning (fringe);
4. balancing conflicting interests or handling aporias (plunge, conflict, balancing).

Table 1 shows a summary of the various aspects of being on “the edge of medicine” discussed so far.

**Table 1:** A summary of some important meanings of “the edge of medicine,” some examples, the corresponding key issues and challenges as well as the role of philosophy and ethics

Meaning of “on the edge”	Example	Key issue and Challenges	Role of Philosophy and/or Ethics
**Border** On the border of what belongs to or counts as medicine	Expansion of concepts of disease, illness, or sickness. Between esthetics and ethics (Cosmetic surgery) Between healthcare and home care Between professionals and patients/relatives (roles) Between covered and non-covered services (dentistry) Between medical and “non-medical conditions”: • Sports medicine • Female genital mutilation • Male circumcision	**Demarcation** (of subject matter) Differentiating between what is disease (illness or sickness) and not, what belongs to the goals and tasks of the health professional and the health care system and what is more appropriately handled by others, and when the health services do more good than harm.	Defining essence or goal Revealing diagnostic creep, overdiagnosis, overtreatment, medicalization Clarify concepts (disease, aging, autonomy, coercion) Clarify the relationship between professionalism and ethics
**Margin** (of life): Between life and death (non-existence)	Neonatology Palliative care Physician assisted suicide Euthanasia	Demarcation (of existence). Defining the tasks of medicine at the margins of life	Setting limits (to existence) Defining key concepts, such as life, death, person, pain, moral status
**Brink**, Verge	The application of the knowledge and remedies of advanced medicine in areas of austerity Global health emergencies Health services to refugees and asylum seekers • DNA-testing, ethics and migration • Age determination in refugee children • Refugees’ access to health care • Domestic violence among asylum seekers	Relevance • Are our approaches in medicine relevant for other contexts? Jurisdiction • How far does the jurisdiction of our health care system go?	Reflecting on cultural and social contingency, universality Demarcation Reflection on goals Emergency bioethics
**Frontier** Forefront	Forefront of research New technologies • Gene editing, gene drives • DtC genetic testing Experimental treatments Validation of personalised medicine AI-based diagnosis and treatment decisions Medical enhancement	Conception Expansion Demarcation Cost containment Resource allocation	Clarifying concepts (human being, natural, therapy, knowledge, information, responsibility) Clarifying goals Analysing analogies Providing methods for knowledge production and assessment (epistemology).
**Fringes**	Conceptual and moral grey zones	Vagueness (conceptual) Relevance (moral)	Defining and handling: • Vagueness • Relevance
**Plunge**, abyss	Situations without moral resolutions	• Culpability, blame, shame • Moral distress • Residue, remorse • Tragic choices	Defining and handling: • Culpability, shame • Moral distress • Residue (moral) • Tragic choices
**Conflict**	Moral dilemmas Moral failure Moral disagreement Catch-22-situations.	Disagreement Conflicting perspectives	Conceptual clarification Critique of perspectives and arguments Brokering
**Balancing** between unacceptable situations or conditions. Finding **optimum**, panacea	Mammography screening Reject and retake of medical images	Judgement, balancing, Quality, optimization	Finding ways to balance interests, perspectives, and concerns Quality assurance

### The tasks of philosophy and ethics

As I have tried to illustrate, there are ample tasks for philosophy and ethics (together with medicine and other disciplines, of course). One of the tasks common for several of the notions of “the edge of medicine” is the *clarification of concepts*. This goes both for descriptive and normative concepts. Several of the challenges in medicine (as in general) stem from the fact that we strive for another level of precision than the concepts we use allow for. In the clarification of the concepts we use to describe and discuss the issues “on the edge of medicine” lies a deeper understanding of the phenomena we encounter, either they are disease, autonomy, euthanasia, pain, or coercion. However, the examples from “the edges of medicine” show that this task has become ever more challenging, as biomedical technology alters the basic phenomena of human beings, such as aging, life, death, and “the human being” itself. Hence, we need not only to clarify our concepts, but also to shape them.

Accordingly, oftentimes clarification of concepts and theories are not sufficient for the tasks on “the edge of medicine.” We may need *new concepts and new theories*. One example is in biobank research, where biological material does not fit with the basic conceptions of the Roman law (things, persons, and actions) [82]. Biological material is neither things, nor persons, or actions which are the traditional objects for regulation. It is like platypus, difficult to classify [83]. The same goes for health information. We may need new analogies, concepts, and new theories, that address the new challenges that we face on the edge [84]. In particular, we need concepts and theories on personhood, parenthood, consciousness, empowerment, nature, health, disease, illness, justice and many more.

Correspondingly, *clarifying the assumptions and premises of our arguments, methods, and decisions* is equally important. A wide range of assumptions are made in evidence production that are not true [85], not relevant [86], or at least are strongly biased [87]. Correspondingly, premises of ethical arguments oftentimes are taken for granted without further inquiry or discussion [88].

Another important task for philosophy and ethics is to *conceptualize and handle uncertainty*, vagueness, ambivalence, indeterminacy, as well as moral residue and regret (10.1007/s10516-021-09573-4). These are hard tasks of great importance both in the epistemology and ethics at the core of medicine, and even more so on the edge of medicine where new modes of knowledge production with unprecedented moral implications implores philosophical and ethical exploration.

One equally important task of philosophy and ethics (related to conceptual clarification) is to *reveal “the emperor’s new clothes*.” In times of huge hype and high hopes it becomes crucial to critically investigate hyped promises, undermine false claims, and avoid false hopes [89]. One illustrating example is found in Precision Preventive Medicine where it was claimed that using a risk score for coronary heart disease (CHD) based on large-scale, genome-wide, and targeted genetic association “substantially advances the concept of using genomic information to stratify individuals with different trajectories of CHD risk and highlights the potential for genomic screening in early life to complement conventional risk prediction.” [90] (p.1883) However, on closer scrutiny it is found that the approach “was only minimally better than the C index of individual cardiac risk factors, such as cholesterol or blood pressure.” [91]. While it was boldly claimed that the risk prediction for common diseases using polygenic risk scores was “a giant leap for gene-based diagnostic tests” [92] more intimate inquiry revealed that “[w]ith such poor test performance characteristics, the use of this genetic risk score would not lead to the right treatment of the right patient at the right time.” [91]. This is but one illustrating example.

One other crucial task for philosophy and ethics is to *identify trends and reflect on their implications*. For example, trends in new diagnostics add radically to the traditional trend where human judgement has been excluded more and more from the assessment of disease. First, the narrative of the patients was reduced or excluded by the introduction of various instruments, such as the stethoscope. Thereafter, the physician’s subjective interpretation of the symptoms, signs, and measurements were excluded by introducing lab tests and genetic tests being independent of physicians’ unreliable interpretations. Now we are on the verge of outsourcing the overall assessment of the meaning of the many measurements for this particular patient to technology, such as artificial intelligence. On the frontier of medicine we distrust ourselves and put our faith in incomprehensible black-boxes. We have outsourced the examination of the patient, the interpretation of the results, and the judgment of what they mean for this particular person, to technology in what may be called “externalized artificial diagnosis and decision.” This poses the question of what is left for health professionals. To take responsibility? This illustrates how *foresight, thought experiments, and critical scenario (or vision) assessment* are important tasks for philosophy and ethics.

Clear and appropriate concepts and theories as well as true premises, valid methods, relevant theories, and sound arguments are all needed for one of the most important tasks for philosophy and ethics on “the edge of medicine,” i.e. the task of *demarcation*. To clarify what is the subject matter of medicine, what is morally relevant, and what belongs to the jurisdiction of medicine, we need clear measures for demarcation.

Issues of demarcation are certainly not easy. However, we were not attracted to philosophy because of its easiness. One of the reasons why the task of demarcation is so difficult is because it relates to or even hinges on another basic question in medicine: “what is the goal of medicine?” Having a clear goal of medicine makes it easier to differentiate and navigate on the edge of medicine. However, the issue of medicine’s goal reaches back to one of the most profound questions in philosophy, i.e., “what is the good life?” – *what is goodness*?

At the same time, what we do at the edges of medicine frames and forms the core of medicine, its goals and values. Never has this been more relevant than in the increasing use of procedural approaches in ethics. We face with the basic challenge of whether to demarcate, define, and handle issues “at the edge of medicine” by referring to the essence or core, or whether we have to stick to procedures, as it is considered useless to refer to any substance or essence of medicine, as the values in medicine are formed by the distinctions that we make at the edges.

Moreover, we also need to reflect on the role of philosophy and medicine for what happens at the edge of medicine. We tend to profit from the expansion of medicine. As excellently phrased by Nancy King: “Ensuring that developments at the edge of medicine and science fall within scientific medicine’s grasp is a primary goal of academic bioethics because it ensures employment for bioethics scholars.” [93]. The developments on the edge of medicine provides more funding for bioethics and “both expand the scope and authority of bioethics and create more silos of narrowly focused expertize.” [93].

## Discussion

### Reforming philosophy and ethics

Medicine does more than just providing philosophy and ethics with hard tasks and ample employment. The issues and challenges on “the edge of medicine” confronts philosophy and its traditional approaches as well. As such the tasks on the edge of medicine can resuscitate, reform, and revitalize both ethics and philosophy in general.

New technologies challenge basic philosophical conceptions such as personhood, consciousness, embodiment, and empowerment. For example, computer-brain-connections and the potential to download the content of human brains or to generate self-consciousness on computers, challenges basic conceptions in philosophy. Hence, the new technologies of the edge of medicine and science put philosophy on its edge. Traditional core concepts concepts, such as “humanity” and “natural” are at play.

New challenges pose new questions needing new perspectives and approaches. As already pointed out: emergent technologies do not only revitalize the question of “what is a human being?” but they also pose the crucial question “what does it meant to be a human being?” and “what should we make a human being be?” Such questions may demand new and broader perspectives than we have developed. We may need a philosophical enhancement (not to be confused with intellectual enhancement or artificial philosophy). As Marquis de Condorcet said many years ago: «the perfectibility of man is unlimited» (1794). However, we tend to confuse better with more: more intelligence, more oxytocin, longer life – or as in Goethe’s Faust: more land [94]. We need to think better.

### Edgy issues

I have taken the use of the metaphor “the edge” as a fact and a premise in this article. However, why are we so challenged by “edgy” things? Metaphor and language theory may give us some answers. In the wake of Lakoff we may think of edges as places where we lose oversight, where we might fall over, or where we need to balance, i.e., edges are dangerous places [95]. At the same time the phrase “the cutting edge” both refers to the sharp effect or quality of something and the foremost part or place.^3^ One of the challenges in medicine is that the foremost technologies may have neither the sharp effects nor the best quality in terms of outcomes. Nonetheless, the edge-metaphor appears to address something deeply human, that is, the combination of despair and hope, of dissipation and formation of norms, and of the lack of control and the potential gaining of new governance. Hence, the edge metaphor touches upon something basically human, and the point here has been to study how this plays out in medicine and the contributions of philosophy and ethics in circumstances appearing to be edgy.

Certainly, many of the identified tasks for philosophy and ethics are not new. Clarification of concepts, premises, and arguments are well-known tasks. However, as I have tried to emphasize by the various interpretations of “the edge of medicine” and the examples, the traditional tasks become ever more crucial as the traditional phenomena, concepts, and normative systems used to address the challenges in medicine are themselves undermined. Additionally, there are completely new tasks in shaping and defining the human being ontologically, epistemically, and ethically.

### Limitations of the approach

Certainly, this investigation has numerous limitations. For example, there are many conceptions of “the edge of medicine” that I have not covered in this article. One example could be “outskirt.” Another could be waterfall and up- and downstream problems [96]. However, the objective of this study has not been to be exhaustive. There are also overlaps between several of the meanings of “edge” discussed in this article, e.g., between balance and conflict. Hence, the conceptions of this study are not mutually exclusive either. Moreover, the conceptions could have been ordered and analyzed in many other and different ways. However, the typology is not meant to carve nature at its joints, but only used to highlight the important role that philosophy and ethics play.

Additionally, there are many important issues that I have not been able to address in this study. Performing medicine on the edges of civilization [97] is but one example. This does of course not mean that philosophy and ethics does not have an important role to play here as well. Part of this is covered by disaster bioethics, and other contributions may be very valuable.

Other fields than philosophy and ethics may of course also have important inputs on all the topics and challenges discussed here, such as all the social sciences, history, and literature, or the medical humanities in general in addition to medicine itself, of course. As indicated at the outset, addressing such approaches is beyond the scope of this article, and has partly been done elsewhere [98–101].

Moreover, some have investigated the role of philosophy in specific areas of medicine in more detail [102], while others have scrutinized the role of philosophy for science more generally [103]. Both attempts are very much in line with the present study. However, they fall outside the three key questions addressed here. This does not mean that they are unimportant. As pointed out by Albert Einstein: “A knowledge of the historic and philosophical background gives that kind of independence from prejudices of his generation from which most scientists are suffering. This independence created by philosophical insight is—in my opinion—the mark of distinction between a mere artisan or specialist and a real seeker after truth.” (Albert Einstein, Letter to Robert Thornton, 1944).

Furthermore, I have not distinguished sharply between the tasks of philosophy and ethics. The phrasing in this study has been inclusive where several readers would prefer exclusiveness. I fully accept this objection. Another objection is that philosophy and ethics has not had any significant impact on the development of medicine so far, so there is little reason to expect any substantial influence in the future. In short, what happens in or at the edge of philosophy (or ethics) has little effect on what happens at the edge of medicine. While I fully acknowledge the relevance of this objection, it is important to notice that we do not know how the development of medicine had been without philosophy and ethics (counterfactually). Moreover, some changes in medicine, e.g., the turn from paternalism to autonomy, come at least in part from philosophy and ethics. And of course, philosophy and ethics may significantly increase its impact in the future.

Yet another issue that has not been addressed here is the negative role of philosophy and ethics. There may of course be lots of waste in the field of philosophy and ethics, as there is in clinical medicine and in the life sciences [104]. Additionally, I have not discussed the relationship between the goals of medicine and of philosophy either. These are topics for future studies.

### Where do we go from here?

One may of course be disenchanted, as many of the issues we discuss as being on “the edge of medicine” today have been on the agenda since the inception of philosophy of medicine and bioethics. Already 30 years ago Dan Callahan wrote in his book “What Kind of Life: The Limits of Medical Progress” that “[w]e are only now beginning to see that we cannot have it all,” [105] (first version 1990). And before that he was preoccupied with setting limits to medicine in an ageing society (by defining its goals) [106]. The same challenges are as pressing – if not more pressing – today. Instead of resigning, this clearly is an impetus to work even harder and differently. Additionally, a wide range of practical measures have been taken to limit, balance, and demarcate medicine, as shown in Table 2.

**Table 2:** Initiatives to provide appropriate care (avoiding underuse and overuse). Based on [107, 108]

Name of initiative, country	Description, year	Link
NICE „DoNotDo “Database, UK:	Savings and Productivity, 2006	http://www.nice.org.uk/
Choosing Wisely (AIMB), USA +	Reducing waste, 2012	http://www.choosingwisely.org/
Slow Medicine, IT	Appropriate care, 2013	http://www.slowmedicine.it/
Preventing Overdiagnosis, UK + USA	Reducing overdiagnosis, 2013	http://www.preventingoverdiagnosis.net/
Lown Institute: Right Care Movement, USA	Appropriate care, 2013	http://lowninstitute.org/take-action/join-the-right-care-alliance/
Smarter Medicine, CH	Appropriate care, 2014	http://www.smartermedicine.ch/
Prudent Health Care, Wales-UK	Prodent care, 2016	http://www.prudenthealthcare.org.uk/
Wiser Healthcare, AUS	Research collaboration for reducing overdiagnosis and overtreatment	https://www.wiserhealthcare.org.au/

Certainly, I have not presented the solutions to the many challenges that we face with on “the edge of medicine” or discussed which approaches in philosophy and ethics that would be most appropriate or suitable to address these challenges. That is beyond the objective of this study. Here the aim has to been to 1) investigate various meanings of “the edge of medicine”, 2) to scrutinize some of the challenges addressed with these meanings, and 3) to illustrate and investigate the role and tasks of philosophy and ethics to address these challenges. While many of the tasks are not new, some are revitalized, reperspectivized, and reactivated. Others are brand new and challenge the foundations of traditional philosophy and ethics.

With respect to handling edges, we may learn from mountain climbers. In the Oscar-winning documentary Free Solo (2019) the National Geographic film-maker Jimmy Chin expresses his concern with filming Axel Honnold several years preparing for and doing his free soloing of the Nose of El Capitan in Yosemite: “If you are pushing the edge, eventually you find the edge.” (Jimmy Chin, Free Solo, 2019, 49:10 min). Alex Honnold on his part claims that: “There is the thing you just have to push because it is that cool.” (Alex Honnold in Free Solo, 2019). In medicine, we deal with other people’s lives, and we need to be tempered and deliberate and not “that cool.”

## Conclusion

I started out this study asking three questions:

1. What does “the edge of medicine” mean in contemporary debates on modern medicine?
2. What are the challenges “on the edge of medicine” (in these various meanings of “on the edge”)?
3. How can philosophy and ethics contribute with addressing these challenges?

I have tried to show that “the edge of medicine” has many meanings, such as: Border; Margin (of life); Frontier; Forefront; Fringes; Plunge (abyss); Brink (verge); Conflict; and Balancing. These meanings seem to address four basic challenges, i.e., setting limits, keeping control, make meaning, and handling conflicts or aporias. In analyzing the various meanings of “the edge of medicine” and illustrating them with examples, I have identified a wide range of important and urgent tasks for philosophy and ethics.

In analyzing the many tasks I have tried to identify a range of overarching chores for philosophy and ethics, such as: 1) clarifying concepts; 2) clarifying assumptions and premises of arguments, methods, advice, and decisions; 3) elaborate new concepts and new theories; 4) conceptualize and handle uncertainty; 5) reveal “the emperor’s new clothes;” 6) identify trends and reflect on their implications; 7) demarcation; and 8) reflecting on goodness.

This does not only underscore and invigorate existing roles of philosophy and ethics but also that completely new tasks are needed at “the edge of medicine.” There is a lot of work to be done – for the improvement of health and wellbeing of living beings – now and in the future. And for shaping and defining the future that is so much formed by “the edge of medicine” in times of gene editing, precision medicine, and artificial intelligence.

## Data Availability

There is no data beyond what is published in this article (references).

## Abbreviations

ALS: Amyotrophic lateral sclerosis
DSM: Diagnostic and Statistical Manual of mental disorders
DtC: Direct-to-Consumer
ESPMH: European Society for Philosophy in Medicine and Health Care
ICD: International Classification of Diseases

## Footnotes

1 The edge metaphor can also bee seen in the SARS-COV-2 pandemic, for example in the news headlines: “Nationwide coronavirus surge pushes hospitals to the edge” https://www.cbsnews.com/news/covid-surge-hospital-staff-nationwide/ CBS News November 252,020.

2 The Scripps Research Institute has an annual Beutler Lecture on the Edge of Medicine where they mainly present new technologies or research breakthroughs, such as “The gut microbiota and childhood undernutrition: Looking at human development from a microbial perspective.” https://www.scripps.edu/newsandviews/e_20140210/updates.html

3 See for example Merriam Webster’s Dictionary: https://www.merriam-webster.com/dictionary/cutting%20edge
